# Surgical drainage of severe Vogt-Koyanagi-Harada-like syndrome secondary to immunotherapy

**DOI:** 10.1016/j.ajoc.2026.102563

**Published:** 2026-03-13

**Authors:** Paul Villain, Gaetan Naux, Lucas Bellot

**Affiliations:** a Department of Ophthalmology, Rennes University Hospital – Pontchaillou Hospital, 2 Rue Henri Le Guilloux, 35000, Rennes, France; b Department of Ophthalmology, Lorient Hospital, 5 Av. Choiseul, 56322, Lorient, France

## 1 Introduction

Immune checkpoint inhibitors (ICIs) have revolutionized the treatment of metastatic melanoma, significantly improving patient survival. However, these therapies are also associated with a wide range of immune-related adverse events, including rare but potentially vision-threatening ocular complications. We report a unique case of severe Vogt-Koyanagi-Harada (VKH)-like syndrome secondary to combined ipilimumab and nivolumab therapy, characterized by persistent choroidal detachment refractory to medical management and requiring surgical intervention. This case highlights both the diagnostic and therapeutic challenges of immune-related ocular inflammation and the importance of multidisciplinary care in such complex presentations. To our knowledge, there are no reported cases in the literature describing persistent choroidal detachment resistant to anti-inflammatory treatment.

## 2 Observation

We present the case of a 52-year-old male caucasian patient who consulted the ophthalmology emergency department due to bilateral vision loss lasting for two weeks, without associated pain. His medical history included a nodular cutaneous melanoma, pT4b of the right thigh, initially metastatic to the thoracic region, lymph nodes, right inguinal region, left thigh, spinal muscles, left trapezius, and left anterior temporal lobe of the brain, diagnosed on cerebal MRI and PET scan, intraocular or orbital metastases were ruled out. This melanoma was diagnosed four months prior and was BRAF wild type. Treatment consisted of dual immunotherapy validated in a multidisciplinary consultation meeting with ipilimumab 5mg/ml – 1mg/kg and nivolumab 10mg/ml-3mg/kg initiated at diagnosis (one injection of nivolumab every 15 days combined with one injection of ipilimumab monthly), with the last dose administered one month before presentation. Since starting treatment, the patient also developed secondary diabetes, autoimmune thyroiditis, and extensive vitiligo. He had no prior ophthalmologic history and no past medical history.

## 3 Clinical case

**Fig. 1:**
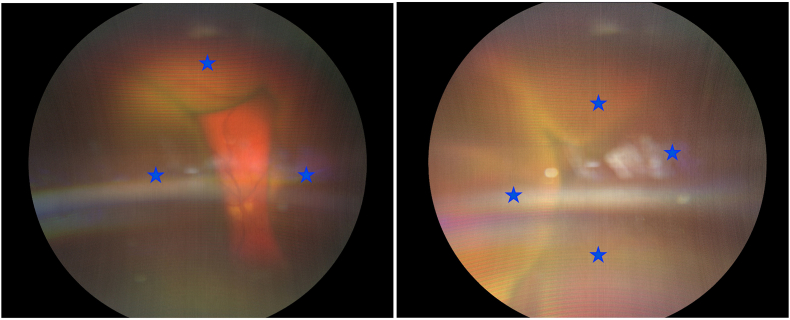

Upon ophthalmologic examination, visual acuity was limited to “positive light perception” in both eyes. Intraocular pressure was normal and symmetrical. Anterior segment examination revealed white eyes with cellular Tyndall graded 3+, fine retrodescemetal precipitates, 360° synechiae between the iris and crystalline lens in both eyes and onset of posterior subcapsular cataract. Fundoscopy showed multiple choroidal detachments extending close to the posterior lens, with no visualization of the posterior pole (Fig. 1: Fundus photographs during the initial evaluation, star: voluminous choroidal detachment).

**Fig. 2:**
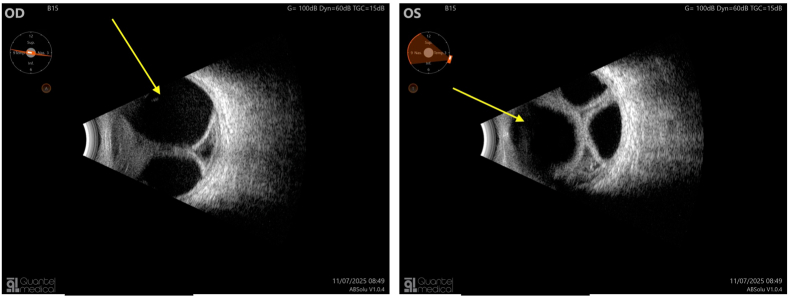

B-mode ultrasound confirmed the presence of a severe choroidal detachment with kissing sign without a visualized intraocular mass (Fig. 2: B-mode ultrasound confirmed the presence of a severe bilateral choroidal detachment with kissing sign, arrow: voluminous choroidal detachment).

Therapeutic management included topical treatment for anterior segment inflammation, combined with three intravenous boluses of 1 g of solumedrol over three days. This was supplemented with sub-tenon injections of triamcinolone 40mg/ml and two injections of infliximab 5mg/kg.

Due to the absence of favorable progress, bilateral rescue choroidal drainage surgery was planned 10 days after the initial consultation.

Bilateral ophthalmic surgery combined phacoemulsification with intraocular lens implantation, choroidal drainage, vitrectomy, and intraocular silicone tamponade at the same time. The first surgical step was phacoemulsification with lens implantation. Following this, anterior chamber infusion was established, and a 3 mm full-thickness scleral incision was made 6 mm from the limbus at the 9 o'clock position (area where choroidal detachments were most significant preoperatively), allowing the extrusion of subchoroidal fluid and complete resolution of the choroidal detachments. Finally, a 25-gauge pars plana vitrectomy via a triple transconjunctival approach. In view of the presence of per-operative hypotonia and a post-operative hemorrhagic risk, silicone tamponade was performed. The procedure was subsequently carried out on the contralateral eye.

The postoperative course was satisfactory, with corrected visual acuity recovery of 0.25 P10 in the right eye and 0.7 P5 in the left eye. Immunotherapy, identified as the cause of this condition, was discontinued due to adequate metastatic control.

**Fig. 3:**
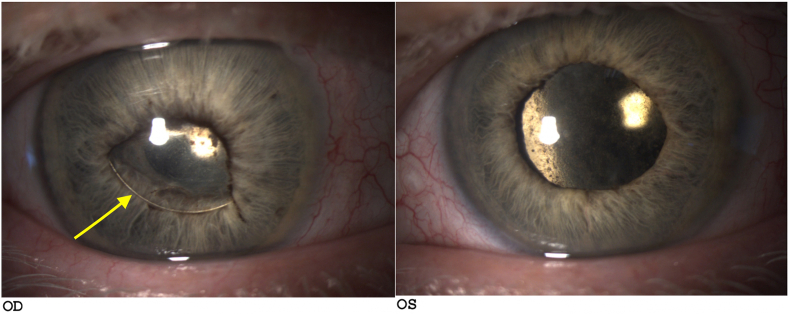

**Fig. 4:**
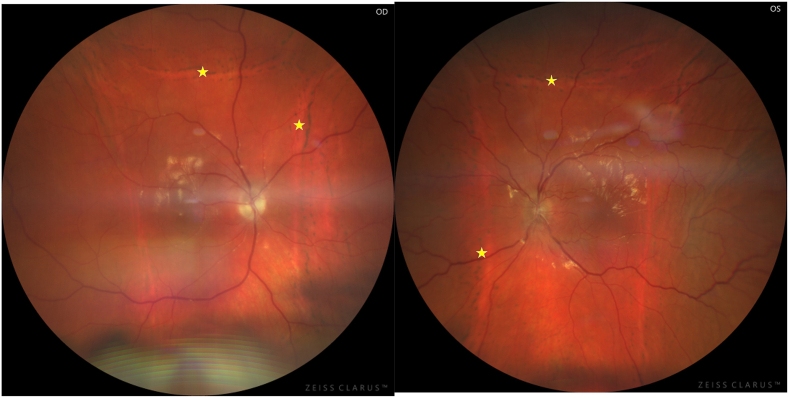

One month postoperatively, the right eye exhibited a sulcus-tilted lens implant with iris synechiae (Fig. 3: Photographs of the anterior segment after surgical management; arrow: sulcus tilted lens in the right eye), a flat retina without recurrence of choroidal detachment, and pigmentary changes at the edges of the choroidal detachments (Fig. 4: Fundus photographs after surgical management, star: pigmentary changes at the edges of the choroidal detachments). The left eye displayed an intracapsular lens implant with 360° iris synechiae, a flat retina without choroidal detachment recurrence, and pigmentary changes at the edges of the choroidal detachments.

**Fig. 5:**
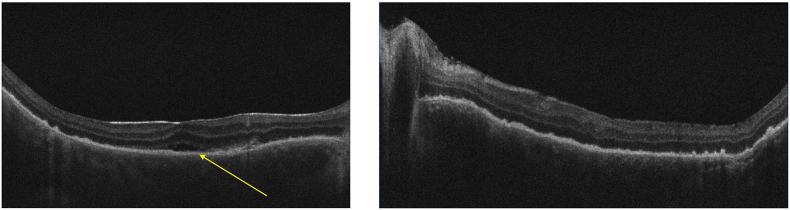

Macular OCT post-surgery revealed retinal atrophy associated with a micro-serous retinal detachment in the right eye, which resolved within one month (Fig. 5: Evolution of macular OCT in the right eye one-month post-surgical management, arrow: micro-serous retinal detachment).

During follow-ups, visual acuity remained stable, intraocular pressure was within normal limits, and there were no changes in the anterior segment of either eye.

**Fig. 6:**
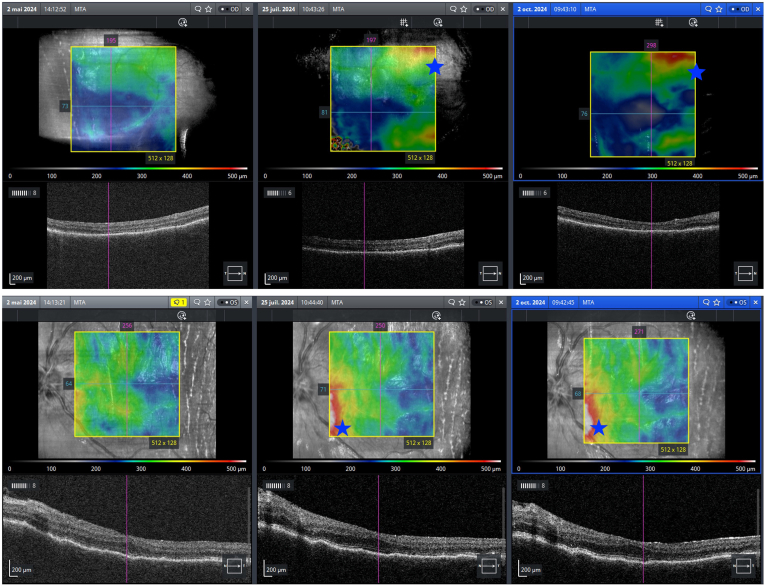

Six months postoperatively, macular OCT revealed peripapillary retinal thickening in both eyes, raising suspicion of inflammatory recurrence (Fig. 6: Evolution of macular OCT six months after surgical management, star: peripapillary retinal thickening in both eyes).

**Fig. 7:**
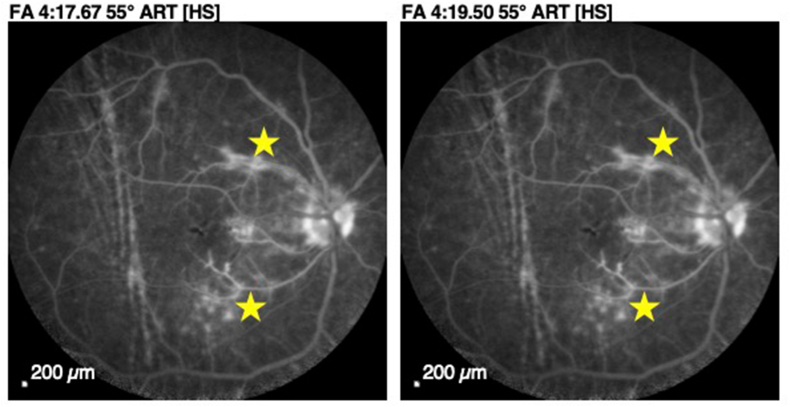

**Fig. 8:**
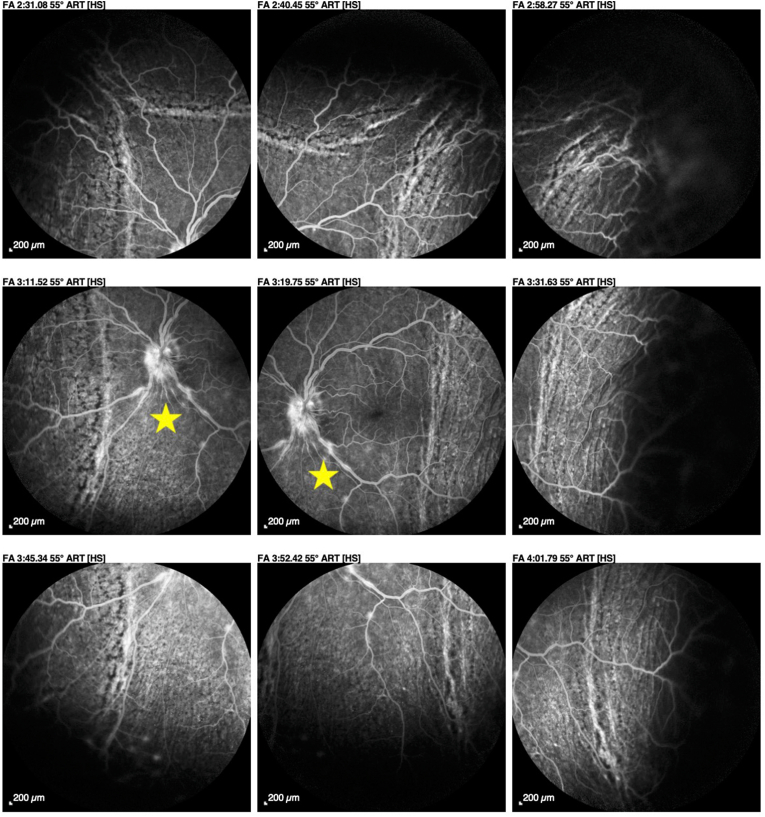

Choroidal fluorescein and indocyanine green angiography demonstrated bilateral papillary leakage associated with non-occlusive vasculitis predominantly affecting the macular arterial trunks on the right and inferior arterial trunks on the left (Fig. 7: Fluorescein angiography of the right eye showing large vessel vasculitis in both eyes, star: non-occlusive vasculitis predominantly affecting the macular arterial trunks & Fig. 8: Fluorescein angiography of the left eye showing large vessel vasculitis in both eyes, star: non-occlusive vasculitis predominantly affecting the macular arterial trunks).

After national expert consultation and multidisciplinary discussion regarding the patient's prognosis, treatment with tocilizumab was initiated, despite limited scientific evidence.

## 4 Discussion

This patient developed a VKH-like syndrome secondary to immunotherapy for metastatic melanoma, requiring rescue choroidal drainage. The outcome was favorable, with recovery of corrected visual acuity.

Immune checkpoint inhibitors (ICIs) comprise three classes: CTLA-4 inhibitors (ipilimumab, tremelimumab), PD-1/PD-L1 inhibitors (nivolumab, pembrolizumab, cemiplimab, atezolizumab, avelumab, durvalumab), and LAG-3 inhibitors (relatlimab). These are primarily used to treat lung cancer and melanoma. The most frequently reported ophthalmologic adverse events include dry eye, uveitis, and myasthenia.^1^^,^^2^ Uveitis, often bilateral (80%), is typically anterior or panuveitic and severe. The risk of intraocular inflammation varies depending on the drug used.^3^

VKH-like syndrome presents as bilateral panuveitis with choroidal thickening, optic disc edema, and serous retinal detachment. Weeks after the acute phase, limbal and fundus depigmentation (sunset glow fundus) may occur. The onset of complications varies from two weeks to two years after initiating immunotherapy, with most cases occurring within the first six months.^3^^,^^4^

Recent reviews recommend systematic ophthalmologic evaluations before starting immunotherapy in patients with risk factors (e.g., prior uveitis, ocular surgery or trauma, autoimmune diseases, renal insufficiency). Ocular inflammation treatment typically involves corticosteroids and certain immunosuppressants; however, these can reduce immunotherapy efficacy. The benefit-risk balance must be evaluated, and continuation of immunotherapy discussed with the oncologist.

We opted for rescue choroidal drainage surgery. Choroidal drainage techniques have primarily been studied post-filtration surgery. In the literature, drainage is indicated for choroidal detachment with “kissing sign,” shallow anterior chamber, or prolonged detachment with a shallow anterior chamber.^5^ In the literature, the surgical management of exudative retinal detachment with uveitis remains a challenge, and there is no clear consensus on the management.^6^ The literature on surgical intervention is very limited to a few case series^6^, ^7^, ^8^. Galor et al.^7^ suggested surgical drainage with scleral buckle, vitrectomy and gas tamponade in inflammatory exudative retinal detachment that persists for more than 3 months after medical therapy. It can act as an adjuvant to conventional medical therapy and help maintain structural integrity, early vitrectomy may help preserve visual function. In our case, it was the risk of structural damage that led us to implement this therapeutic strategy.

The most described technique involves a sclerotomy performed 3.5 mm posterior to the limbus using a 15° side-port blade, targeting the most prominent choroidal detachment as identified via ultrasound or indirect ophthalmoscopy. The scleral incision measures 1 to 1.5 mm. A 30-gauge needle is sometimes used to inject BSS into the vitreous cavity through the same sclerotomy to facilitate subchoroidal fluid drainage. A standard three-port pars plana vitrectomy is then performed.^9^ Some experts recommend simultaneous vitrectomy to reduce the risk of retinal detachment, giant retinal tears, or proliferative vitreoretinopathy. Others perform vitrectomy only when such complications develop.^9^ Rezende et al. suggest inserting the trocar 7 mm posterior to the limbus instead of the pars plana to avoid retinal damage.^10^ After fluid drainage, thorough inspection of the drainage site using wide-angle visualization or indirect ophthalmoscopy is advised to ensure no perforation or retinal tissue incarceration.^11^ Matlach et al. described a novel choroidal fluid drainage technique using a penetrating diathermy probe. This procedure involves draining choroidal fluid with a 2 mm-tipped penetrating diathermy probe after placing a 20-gauge infusion line in the anterior chamber.^12^

## 5 Conclusion

This case underscores the importance of close monitoring of patients with functional symptoms or risk factors for ocular inflammation, as well as highlighting the potential for severe ocular involvement during immunotherapy treatment.

## CRediT authorship contribution statement

**Paul Villain:** Writing – original draft, Validation, Resources, Methodology, Funding acquisition, Conceptualization. **Gaetan Naux:** Writing – review & editing, Validation, Investigation. **Lucas Bellot:** Investigation, Funding acquisition.

## Patient consent

Written consent to publish this case has been obtained in writing.

## Authorship

All authors attest that they meet the current ICMJE criteria for Authorship.

## Funding

No funding or grant support

## Declaration of competing interest

All authors declare no conflicts of interest.
